# Author Correction: Metabolism of multiple glycosaminoglycans by *Bacteroides thetaiotaomicron* is orchestrated by a versatile core genetic locus

**DOI:** 10.1038/s41467-020-18097-1

**Published:** 2020-08-28

**Authors:** Didier Ndeh, Arnaud Baslé, Henrik Strahl, Edwin A. Yates, Urszula L. McClurgg, Bernard Henrissat, Nicolas Terrapon, Alan Cartmell

**Affiliations:** 1 Biosciences Institute, Newcastle University, Newcastle upon Tyne, NE2 4HH UK; 2 Quadram Institute Bioscience, Norwich Research Park, Norwich, Norfolk, NR4 7UQ UK; 3 Centre for Bacterial Cell Biology, Biosciences Institute, Newcastle University, Newcastle upon Tyne, NE2 4HH UK; 4 Department of Biochemistry, Institute of Integrative Biology, University of Liverpool, Liverpool, L69 7ZB UK; 5 Architecture et Fonction des Macromolécules Biologiques, CNRS, Aix-Marseille University, F-13288 Marseille, France; 6 USC1408 Architecture et Fonction des Macromolécules Biologiques, Institut National de la Recherche Agronomique, F-13288 Marseille, France; 7 Department of Biological Sciences, King Abdulaziz University, Jeddah, 23218 Saudi Arabia

**Keywords:** Polysaccharides, Glycobiology, X-ray crystallography, Microbiome

Correction to: *Nature Communications* 10.1038/s41467-020-14509-4, published online 31 January 2020.

The original version of this Article contained an error in the information provided in the legend for Supplementary Fig. 4D. The text should read: 1 = UA-GalNAc4S; 2 = UA-GalNAc4S incubated with B.theta PBS washed cells for 1h; 3 = UA-GalNAc4S incubated with B.theta PBS washed cell for 24h; 4 = UA-GalNAc4S incubated with sonicated B.theta PBS washed cells for 1h; 5 = UA-GalNAc4S incubated with sonicated B.theta PBS washed cell for 24h; 6 = GalNAc; 7 = GalNAc6S; 8 = GalNAc6S incubated with B.theta PBS washed cells for 1h; 9 = GalNAc6S incubated with B.theta PBS washed cells for 24h; 10 = GalNAc6S incubated with sonicated B.theta PBS washed cell for 1h and 11 = GalNAc6S incubated with sonicated B.theta PBS washed cells for 24.

This has been corrected in both the PDF and HTML versions of the Article.

