# Supplementary material for: Metabolism of multiple glycosaminoglycans by Bacteroides thetaiotaomicron is orchestrated by a versatile core genetic locus
Source: Nat Commun. 2020 Jan 31;11:646. doi: 10.1038/s41467-020-14509-4 (PMC6994673; doi:10.1038/s41467-020-14509-4)
Supplement: Supplementary file 1 — Supplementary Information [file 41467_2020_14509_MOESM1_ESM.pdf]

Supplemental Discussion

BT3328^{PL29} direction of cleavage

Initially a HA dodecasaccharide was labelled at its reducing with a 2-AB fluorescent label. This length was chosen ahead of d.p 10 and 8 to reduce the risk of the tag interfering with activity. It was expected that if BT3328^{PL29} acted from the non-reducing a labelled hexasaccharide or octasaccharide would be produced. If the enzyme acted from the reducing end a labelled tetrasaccharide or disaccharide would be produced. The result, however, revealed that a labelled trisaccharide was exclusively produced and the product profile no longer produced an octasaccharide or tetrasaccharide (Supplementary Figure 2C). These data indicate three things: (i) the enzyme operates from the reducing end (ii) the presence of the label increases affinity for the reducing end (judged by the lack of a tetrasaccharide) (iii) the dodecasaccharide must contain contamination with a d.p.11 or d.p. 13 oligosaccharide with GlcNac at the reducing end. Owing to the unexpected result with the dodecasaccharide we then labelled both an octasaccharide and a decasaccharide with an 7-amino-1,3-napthalenedisulfonic acid (ANDSA) label at their reducing ends. A partial digest revealed BT3328^{PL29} exclusively produced a labelled disaccharide from the labelled substrates, demonstrating BT3328^{PL29} unambiguously acts from the glycans reducing end (Supplementary Figure 2D).

Activity of Δbt3324/Δbt3350 and Δbt3350/Δbt4410 mutants

The out performance of *Δbt3324/Δbt3350* over *Δbt3350/Δbt4410* on CS-A is contradictory to the biochemical activities of BT3324^{PL8} and BT4410^{PL33}. The kinetic data show that BT4410^{PL33} displays significantly reduced rates and accessibility (based on total bonds cleaved) on all sulfated substrates with respect to HA. BT3324^{PL8} on the other hand displays it highest activity against the O4 sulfated substrates CS-A and DS in terms of both rate and bonds cleaved. Therefore, the only way the *Δbt3324/Δbt3350* mutant can out perform the *Δbt3350/Δbt4410* is if the activity of BT4410^{PL33} against CS-A is vastly improved. The only way to make CS-A more like HA, the preferred substrate of BT4410^{PL33}, is to remove the O4 sulfation. The removal of O4 sulfation on CS-A can be performed by the endo sulfatase BT3349$^{4S\text{-}sulf}$, thus enhancing the activity of BT4410^{PL33} against CS-A. Furthermore, BT3324^{PL8} is an exo-acting enzyme, so in the absence of an endo enzyme to create new non-reducing ends, the 'effective' substrate concentration remains the same down to the last

disaccharide unit cleaved. With BT4410^{PL33} being endo processive, it can create multiple non-reducing ends, thus increasing the effective substrate concentration. This, and removal of sulfation, would allow the *Δbt3324/Δbt3350* mutant to out perform the *Δbt3350/Δbt4410* on CS-A. This reinforces the need for endo-exo synergy between BT3350^{PL8} and BT3324^{PL8} in sulfated GAG catabolism.

Mutagenic analysis of the BT3349$^{4S\text{-}sulf}$ binding cleft and active site using CSA

The mutations D33A, H241A and D319A all completely inactivated the protein. H241A is likely the catalytic acid, protonating the leaving group sugar as nucleophillic attack from the formylglycine, on the O4 sulfate group, generates the covalent enzyme-sulfate intermediate. D33A and D319A form part of the calcium binding site, along with H320, and their mutation inactivates the enzyme presumably due to loss of calcium binding. H320A mutation does not completely inactivate the enzyme but could not be quantitatively measured. The calcium binding site has a trigonal bi-pyramid organisation with the planar ligands being Oδ2 of D33, Nε2 of H320 and would be completed by the formylglycine residue. Oδ2 of D319 sits beneath the calcium and the final ligand site is left free for the sulfate to bind and complete the trigonal bi-pyramid geometry. At the 0 subsite T183A had no effect on catalytic activity, Oγ1 of T183 is 3.1 Å away from O6 and thus too far to make a productive polar interaction. The O6 of GalNAc makes a 2.7 Å interaction with D185 and mutation to Ala or Asn caused a serve loss of function, and although still active, their activity could not be quantified. Mutation of W163 to Ala or Phe caused ~5000 fold loss in function demonstrating the requirement for Trp at this position. At the -1 subsite the unsaturated uronic acid is bound inbetween D331, interacting via O2 and O3, and R94, interacting with its carboxylic acid group. The D331A mutant did not express and could not be analysed further. The R110A mutant caused a 500-fold drop in catalytic function but could be significantly recovered by mutation to Lys where only a 20 fold drop in function was observed. N105A, N334A and S242A were all active but again below measurable activity. N105 forms part of the S site and its Nδ2 is 2.7 Å from the sulfate group and may help with charge stabilisation during binding and/or catalysis. S242 makes no direct interactions with the substrate but is near H241 and mutation to Ala may affect the position or pKa of the catalytic acid. N334 also makes no interactions with the modelled trisaccharide but sits below a putative -2 subsite where it could make important interactions.

Mutagenic analysis of BT3333$^{6S-sulf}$

In addition to the interactions described in the main text, the acetyl group of GalNAc potentially interacts with W464 via apolar interactions. The beta face of the GalNAc sits above D339 and Y363 (Figure 5B). The calcium binding site in BT3333$^{6S-sulf}$ adopts a octahedral geometry. The planar ligands are the Oδ1 of D143, D144, N333 and would be completed by the formylglycine catalytic residue. The axial ligands, which complete the octahedral geometry are provided by D332 and the labile sulfate group. The wildtype of BT3333$^{6S-sulf}$ (derived from S83C mutant) displayed limited activity on its substrate GalNAc6S. This could be suggestive of an improper conversion of the Cys at postion 83 to the formylglycine. BT3333$^{6S-sulf}$ also displayed a lower activity in whole cell assays, compared to BT3349$^{4S-sulf}$, suggesting it may be a less efficient enzyme. This makes analysis of the mutants difficult, especially as all but two were inactive (Table S4). Interestingly, although Y363A was completely inactive (using 10 μM enzyme at 37°C overnight), the Y363F mutant appeared to display increased activity, being able to fully desulfate GalNAc6S overnight, when assessed qualitatively. The change of Tyr to Phe may create a more hydrophobic environment with which the beta face of the sugar can interact more favourably.

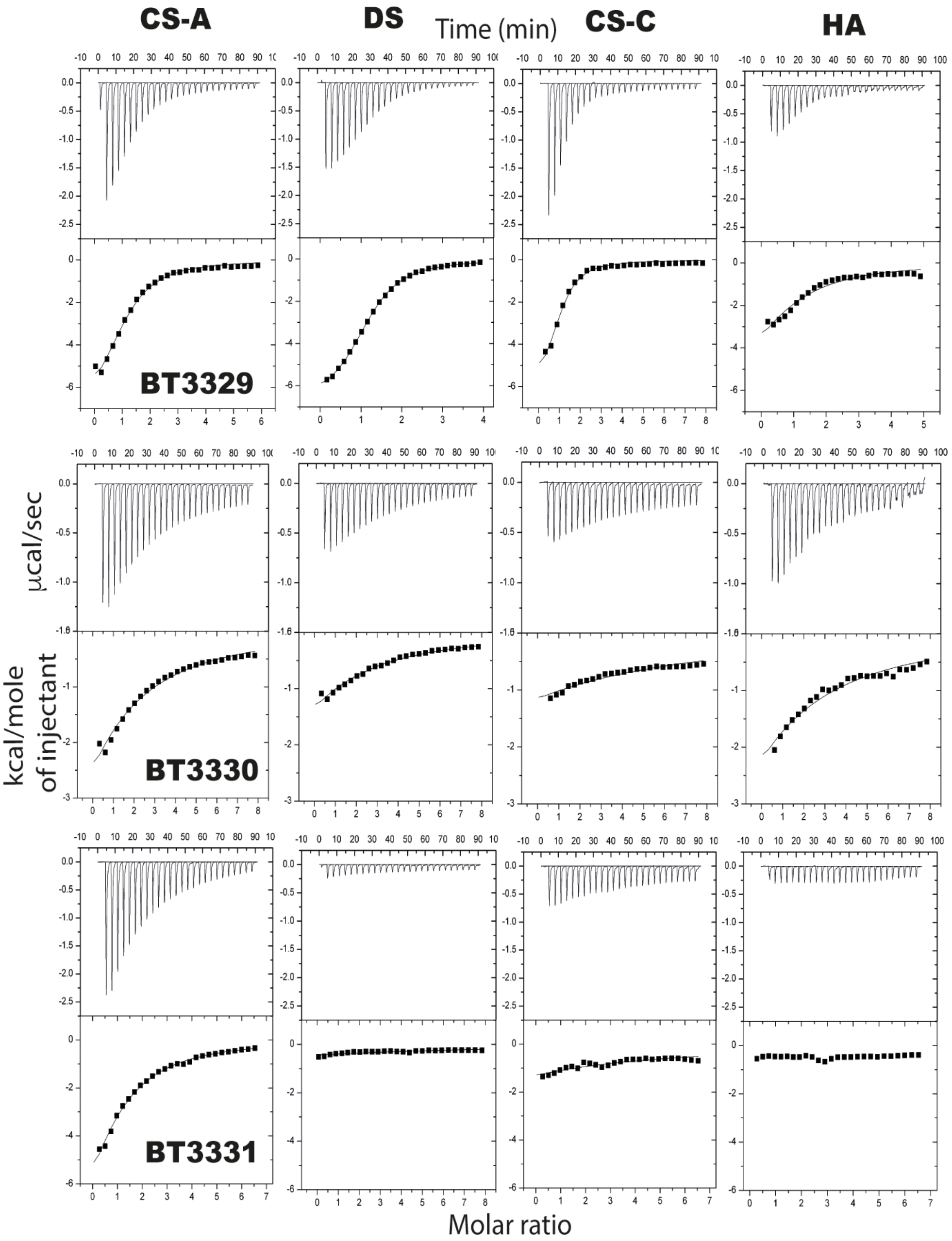

Supplementary Figure. 1. Isothermal titrations of surface glycan binding proteins

All traces are plotted on the same scale for comparison. All traces used 5mg/ml of the relevant glycosaminoglycan, except BT3329^{SGBP} and BT3330^{SGBP} against HA were 3.33 mg/ml was deployed. For BT3329^{SGBP} and BT3330^{SGBP} titrations the protein concentration was 50 μM, whilst for BT3331$^{SusD\text{-}like}$ a protein concentration of 60 μM was used. All titrations were performed in 50 mM Tris pH 8.0 at 25°C.

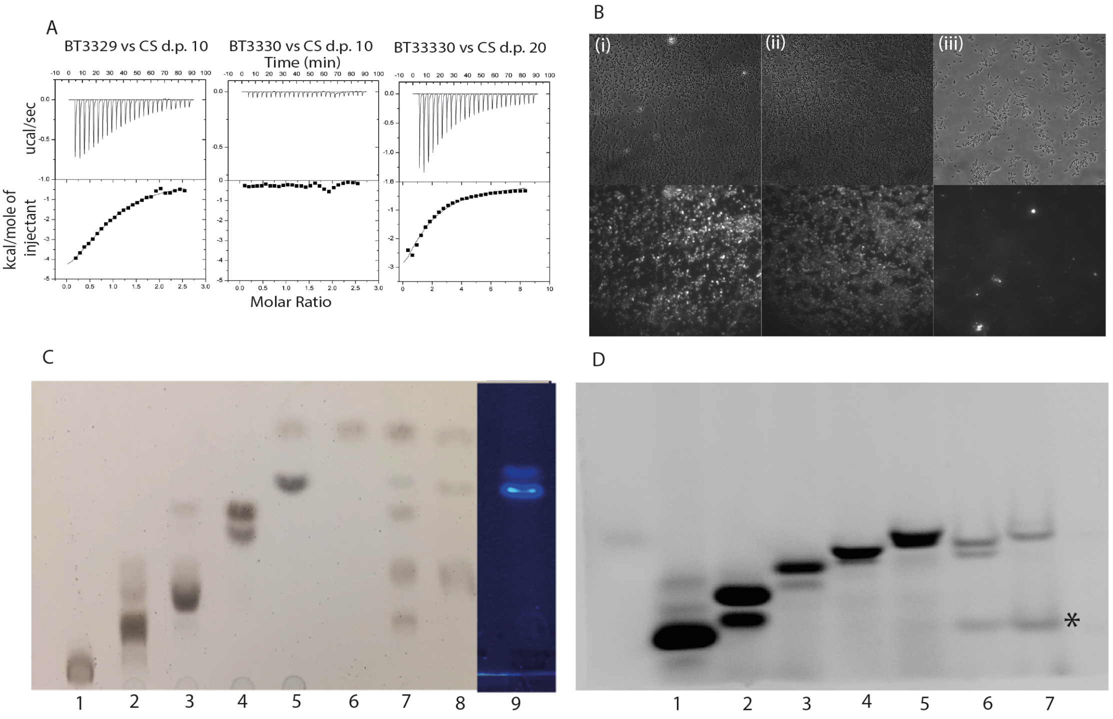

Supplementary Figure 2. Analysis of surface proteins biochemistry and location. (A) ITC analysis of $BT3329^{SGBP}$ and $BT3330^{SGBP}$ binding to CS oligosaccharides of DP10 and DP20; (B) Cell surface accessibility of $BT3329^{SGBP}$ (i), $BT3330^{SGBP}$ (ii), and $BT4657^{PL12}$ (iii) detected by immunostaining. PUL_{Hep}-encoded $BT4657^{PL12}$, which localises to the periplasm, was used as a negative control. (C) TLC of $BT3328^{PL29}$ versus an unlabelled and 2-AB labelled hyalurnonan dodecasaccharide (DP12). 1=DP10, 2= DP8 3= DP6, 4=DP4, 5= DP3, 6=DP2,7=DP12+ $BT3328^{PL29}$, 8= 2-A,B labelled DP12+$BT3328^{PL29}$, 9=2-AB labelled DP12+$BT3328^{PL29}$ exposed to UV light highlighting the location of the labelled glycan product. (D) SDS-PAGE analysis of $BT3328^{PL29}$ versus ASNDA labelled glycans; 1=DP2; 2=DP4; 3=DP6; 4=DP8; 5=DP10; 6=DP8+ $BT3328^{PL29}$; 7= DP10+ $BT3328^{PL29}$. The asterix highlights the presence of a newly created labelled DP2 in the presence of $BT3328^{PL29}$. Source data for panels C and D can be found in source data file as uncropped images.

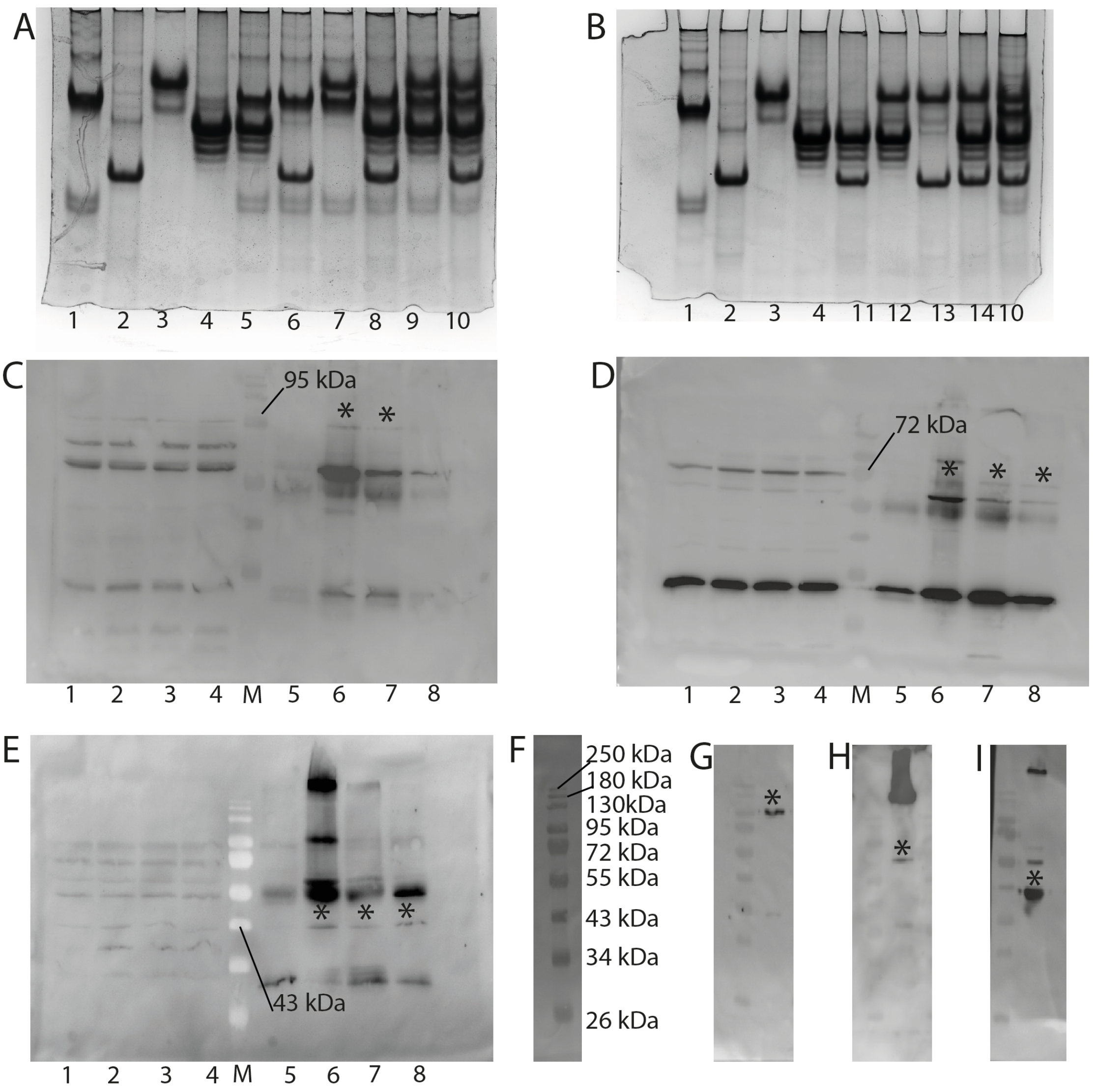

Supplementary Figure 3. Analysis of cell surface protein-protein interactions. (A) and (B) are native PAGE gels. 1=BT3328; 2=BT3330; 3=BT3329; 4=BT3331;5=BT3328+BT3331;6=BT3328+BT3330; 7= BT3328+BT3329;8= BT3328+ BT3330+BT3331; 9= BT3328+ BT3329+BT3331; 10= BT3328+ BT3329+ BT3330+BT3331; 11= BT3330+BT3331; 12= BT3329+BT3331; 13=BT3329+BT3330; 14= BT3329+ BT3330+BT3331. C, D and E are western blots probed for BT3328, BT3329 and BT3330, respectively. 1,2,3 and 4 are the non-immunoprecipitated input controls for the following respective immunoprecipitated samples. 5= co-immunoprecipitation (Co-IP) with non-immunised rabbit IgG**;** 6=co-immunoprecipitation (Co-IP) with anti-BT3328; 7= co-immunoprecipitation (Co-IP) with anti-BT3329; 8 co-immunoprecipitation (Co-IP) with anti-BT3329. M signifies marker. (F) NEB Color-coded Prestained Protein Marker, Broad Range. G, H and I are westerns blots using anit-BT3328, anti-BT3329 and anti-BT3330 antibodies respectively against IMAC purified recombinant protein. * has been placed directly above the protein of interest on all gels. Source data can be found in source data file as uncropped images.

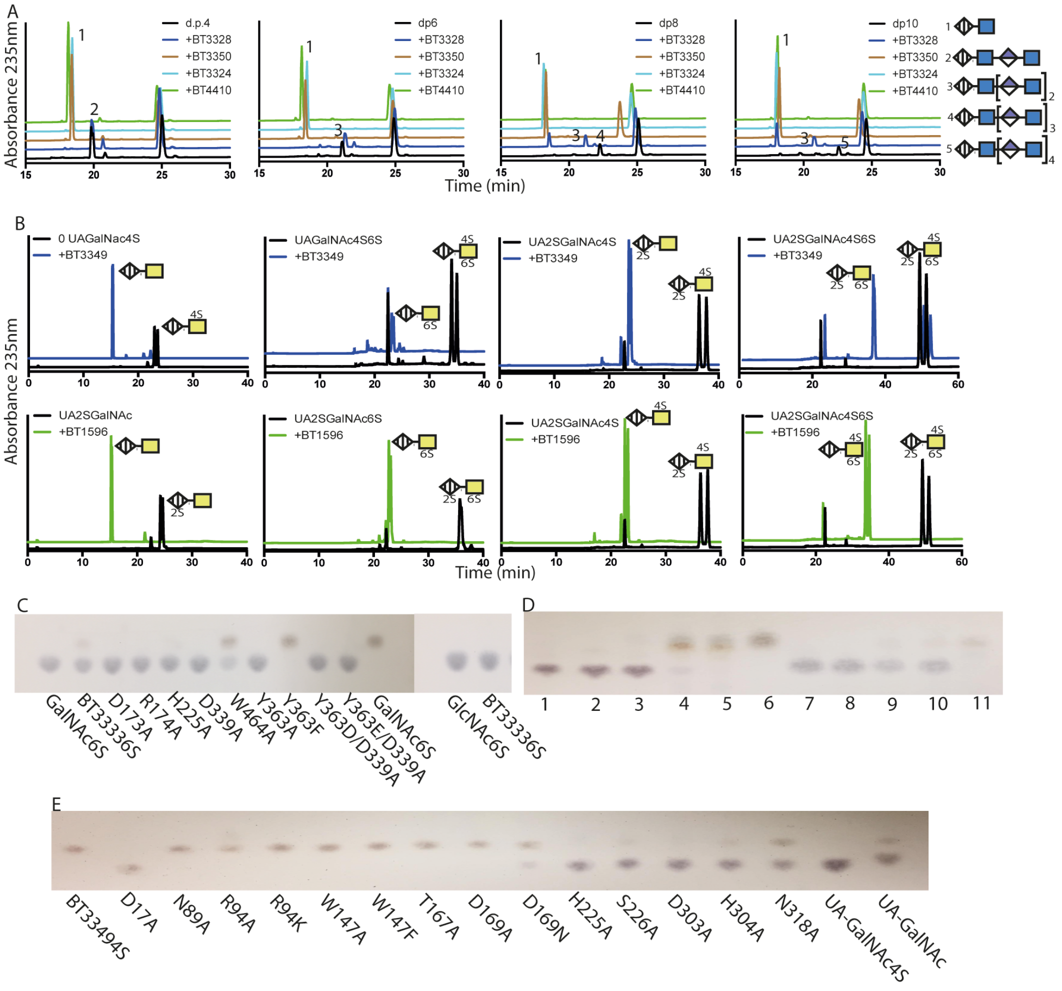

Supplementary Figure 4. Hyaluronan oligosaccharide activity of lyases, sulfation preference of sulfatase and cellular location and sulfatase mutant analysis.

(A) PUL encoded lyases incubated overnight with varying hyaluronan oligosaccharides; (B) Sulfation tolerances of BT3349$^{4S\text{-}sulf}$ and BT1596$^{2S\text{-}sulf}$ against commercial disaccharides; (C) BT3333$^{6S\text{-}sulf}$ and mutants tested against GalNAc6S and GlcNAc6S (D) 1=UA-GalNAc4S, 2=UA-GalNAc4S incubated with *B.theta* PBS washed cell for 1h; 3=UA-GalNAc4S, 2=UA-GalNAc4S incubated with *B.theta* PBS washed cell for 24h; 4=UA-GalNAc4S, 2=UA-GalNAc4S incubated with *B.theta* PBS washed cell for 1h; 5=UA-GalNAc4S, 2=UA-GalNAc4S incubated with *B.theta* PBS washed cell for 24h; 7=GalNAc6S; 8=GalNAc6S incubated with *B.theta* PBS washed cell for 1h; 9=GalNAc6S incubated with *B.theta* PBS washed cell for 24h; 10= GalNAc6S incubated with sonicated *B.theta* PBS washed cell for 1h and 11= GalNAc6S incubated with sonicated *B.theta* PBS washed cell for 24h; (E) BT3349$^{4S\text{-}sulf}$ and mutants tested against UA-GalNAc4S. In the UA-GalNAc standard the upper band is UA-GalNAc whilst the lower band is an unknown contaminant. Source data for panels C, D and E can be found in source data file as uncropped images.

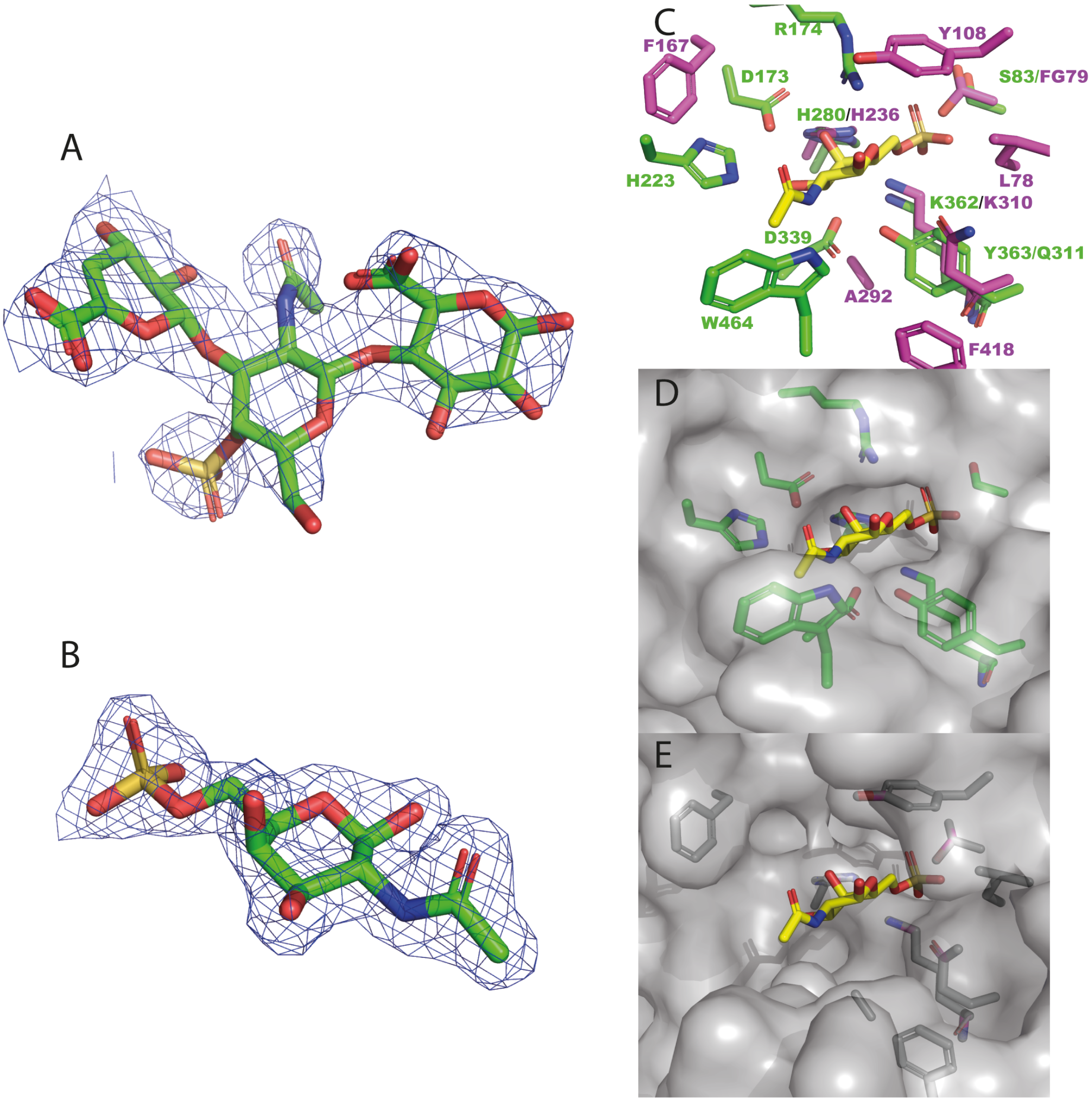

Supplementary Figure 5. Electron density of modelled ligands and overlay of BT3333$^{6S\text{-}sulf}$ with the human GALNS$^{6S\text{-}sulf}$.

A) $2F_{obs}$-F_{calc} composite map of the modelled substrate for BT3349$^{4S\text{-}sulf}$ contoured at 1σ (0.25 e/Å^3); B) $2F_{obs}$-F_{calc} composite map of the modelled substrate for BT3333$^{6S\text{-}sulf}$ contoured at 1σ (0.29 e/Å^3); C) Overlay of the active site interactions of BT3333$^{6S\text{-}sulf}$ and GALNS$^{6S\text{-}sulf}$.The invariant active site residues are S83/FG79, H280/H236 and K362/K310 are shown for comparison to the variable sugar binding site residues. The substrate GalNAc6S is from BT3333$^{6S\text{-}sulf}$; D) Suface representation of BT3333$^{6S\text{-}sulf}$ showing the 'tight' pocket of the enzyme and the interacting residues; E) Suface representation of GALNS$^{6S\text{-}sulf}$ showing the 'open' sugar binding area of the enzyme and the potential interacting residues. The ligand is from the BT3333$^{6S\text{-}sulf}$ structure.

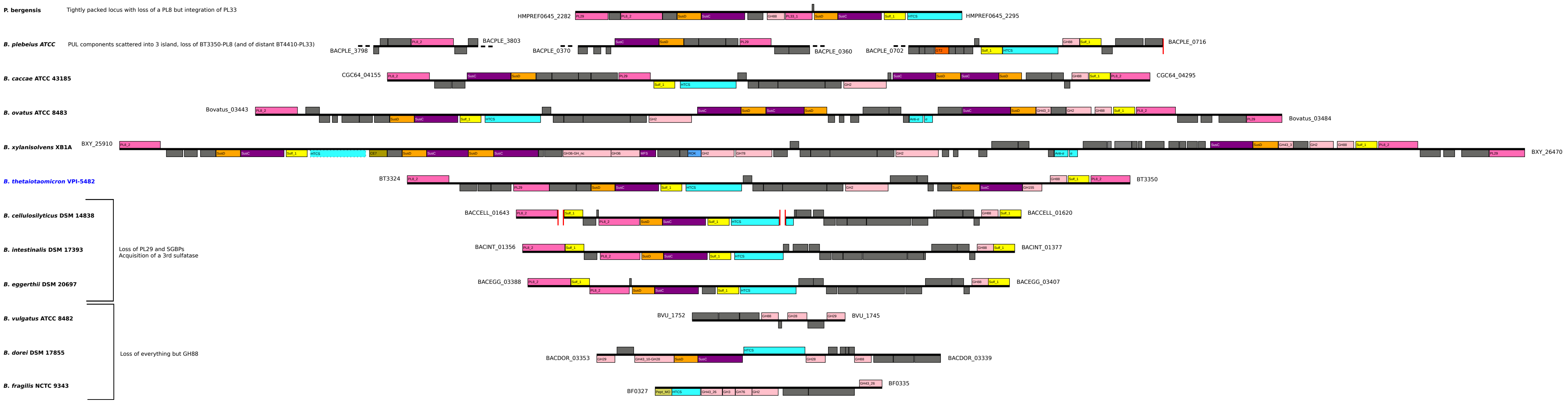

Supplementary Figure 6. Genomic view of *B. thetaiotaomicron* PUL and syntenic regions inselected species
B. thetaiotaomicron and other species tested for growth in this studyare represented below a remote/rearranged but all-inclusive locus in *Prevotella berghensis*. Protein-coding genes are depicted by colored rectangles to highlight the following functional modules: GHs in light pink, PLs in dark pink, sulfatases in yellow, peptidase in gold, SusC transporters in purple, SusD outer-membrane proteins in orange, HTCSs/ECF sigma/anti-sigma regulators in cyan. A missing gene model in *B. xylanisolvensis* surrounded by a dotted line instead. Genes are represented either above or below a central black line according to the coding strand. High distances between *B. plebeius* genomic islands/loci are represented by a dotted central line while in case of incomplete genome assemblies the scaffold ends are indicated by vertical red bars.

Substrate and Protein	*N*	K_A	ΔH	ΔS
**CS-A**				
BT3331^{SusD}	0.89 ± 0.08	(1.09 ± 0.07)x10^{4}	(-1.44 ± 0.07)x10^{4}	-29.8
BT3330^{SGBP}	1.00 ± 0	(4.95 ± 0.20)x10^{3}	(-1.25 ± 0.003)x10^{4}	-25.1
BT3329^{SGBP}	0.96 ± 0.04	(3.58 ± 0.27)x10^{4}	(-9.15 ± 0.47)x10^{3}	-9.86
**CS-C (Sigma)**				
BT3331^{SusD}	NQ	NQ	NQ	NQ
BT3330^{SGBP}	1.00 ± 0	(9.10 ± 0.10)x10^{2}	(-2.6 ± 0.02)x10^{3}	-73.6
BT3329^{SGBP}	0.93 ± 0.03	(5.81 ± 0.05)x10^{5}	(-7.61 ± 0.32)x10^{3}	-3.72
**DS**				
BT3331^{SusD}	NQ	NQ	NQ	NQ
BT3330^{SGBP}	1.00 ± 0	(4.02 ± 0.10)x10^{3}	(-8.13 ± 0.11)x10^{3}	-10.8
BT3329^{SGBP}	1.06 ± 0.05	(1.10 ± 0.02)x10^{5}	(-7.66 ± 0.45)x10^{3}	-2.64
**HA**				
BT3331^{SusD}	NQ	NQ	NQ	NQ
BT3330^{SGBP}	1.00 ± 0	(2.89 ± 0.21)x10^{3}	(-1.69 ± 0.07)x10^{4}	-41.0
BT3329^{SGBP}	1.14 ± 0.04	(1.18 ± 0.38)x10^{4}	(-6.82 ± 2.96)x10^{3}	-4.24
**CS DP10**				
BT3330^{SGBP}	NB	NB	NB	NB
BT3329^{SGBP}	0.81 ± 0.14	(5.30 ± 1.13) x10^{4}	(-7.63 ± 1.80) x10^{3}	-4.01
**CS DP20**				
BT3330^{SGBP}	1.30 ± 0.34	(9.84 ± 0.20) x10^{4}	(-7.65 ± 2.44) x10^{3}	-7.38

Supplementary Table 1. Binding kinetics for the PUL encoded surface binding proteins.

For titrations 50 μM protein was used except for BT3331^{SusD} were 60 μM was protein was utilised. Ligand concentration was 5mg/ml except for BT3329^{SGBP} and BT3330^{SGBP} against HA were 3.33 mg/ml was used. For BT3329^{SGBP} ligand concentrations were floated till N was close to one (CSA, B, C, HA-1.5 mM. 0.9 mM, 1 mM, 1 mM). For BT3330^{SGBP} and BT3331^{SGBP} ligand concentration was fixed at 2 mM and N fixed to 1. Titrations were performed in was 50 mM Tris pH 8.0 at 25°C. NQ indicates binding was too low to be quantified by ITC, whilst NB indicates no binding could be observed by ITC at all. Data were carried out in triplicate and ± equals standard deviation about the mean.

Substrate and Enzyme	K_M (mg ml^{-1})	k_{cat} (min^{-1})	k_{cat}/K_M (min^{-1} mg^{-1} ml)	Total Cleavage (mM/mg)
**CS-A**				
BT3324^{PL8}	0.62 ± 0.16	9345 ± 1441	15072 ± 6180	1.70 ± 0.32
BT3350^{PL8}	0.074 ± 0.02	18631 ± 3840	293,520 ± 140,890	1.42 ± 0.30
BT3328^{PL29}	1.95 ± 0.22	3290 ± 404	1687 ± 404	0.35 ± 0.035
BT4410^{PL33}	>6	-	130 ± 79	0.68 ± 0.1
**CS-C (Sigma)**				
BT3324^{PL8}	>2	-	1499 ± 234	1.55 ± 0.18
BT3350^{PL8}	0.065 ± 0.026	9486 ± 4062	145,661 ± 120,899	0.74 ± 0.24
BT3328^{PL29}	>4	-	175 ± 23	0.72 ± 0.01
BT4410^{PL33}	>4		378 ± 55	1.06 ± 0.17
**CS-C (carbosynth)**				
BT3324^{PL8}	>2	-	1835 ± 135	0.71 ± 0.062
BT3350^{PL8}	0.1 ± 0.036	7778 ± 1365	77,780 ± 34223	0.26 ± 0.13
BT3328^{PL29}	NQ	NQ	NQ	0.11 ± 0.01
BT4410^{PL33}	NQ	NQ	NQ	0.23 ± 0.006
**DS**				
BT3324^{PL8}	0.61 ± 0.07	8578 ± 1439	14062 ± 3,937	1.39 ± 0.27
BT3350^{PL8}	0.021 ± 0.004	13302 ± 5835	633,428 ± 424,397	1.29 ± 0.06
BT3328^{PL29}	NQ	NQ	NQ	0.20 ± 0.031
BT4410^{PL33}	0.71 ± 0.16	260 ± 18	366 ± 117	0.1 ± 0.06
**HA**				
BT3324^{PL8}	>1	-	922 ± 103	2.96 ± 0.21
BT3350^{PL8}	0.16 ± 0.04	3307 ± 916	20,669 ± 10,748	0.76 ± 0.20
BT3328^{PL29}	>4	-	70 ± 2.7	2.14 ± 0.07
BT4410^{PL33}	0.57 ± 0.17	18616 ± 8870	32659 ± 21882	3.21 ± 0.26

Supplementary Table 2. Kinetic parameters for PUL encoded polysaccharide lyases

NQ indicates activity could be observed qualitatively by HPLC or TLC using >10 uM enzyme overnight at 37°C but could not be accurately quantified by spectrophotpmetric methods deployed. All assayed were performed in 100 mM MES or BTP at pH6.0 or 6.5 with 150 mM NaCl at 37°C. All data were carried out in at least triplicate and ± indicates standard error of the mean. Source data are provided in the source data file labelled as kinetics.

Protein	Signal peptide	Cellular location	Comments
BT3324^{PL8}	SpI	Periplasmic	Predicted. N-terminal Methionine is incorrect and should be MIQKS-.
BT3328^{PL29}	SpII	Extracellular surface	Experimental evidence. Ndeh *et al*.
BT3329^{SGBP}	SpII	Extracellular surface	Experimental evidence. This paper.
BT3330^{SGBP}	SpII	Extracellular surface	Experimental evidence. This paper.
BT3331^{SusD}	SpII	Extracellular surface	Predicted.
BT3332^{SusC}	SpI (integral membrane protein)	Outer membrane	Predicted.
BT3333$^{6s\text{-}sulf}$	SpII	Periplasmic	Experimental evidence. This paper.
BT3334^{HTCS}	SpI (integral membrane protein)	Inner memberane	Integral membrane protein
BT3348^{GH88}	SpII	Periplasmic	Predicted
BT3349$^{4S\text{-}sulf}$	SpII	Periplasmic	Experimental evidence. This paper. The predicted N-terminal Methionine is incorrect and should be MINKS-.
BT3350^{PL8}	SpI	Periplasmic	Predicted.
BT4410^{PL33}	SpI	Periplasmic	Predicted.

Supplementary Table 3 Signal peptide and cellular location of CS induced proteins. Signal peptides were determined using the lipop 1.0 and Signal P 5.0 servers. The Kyoto Encyclopedia for Genes and Genomes was used to check the upstream sequence of the studied genes to ensure the correct start methionine was being used.

**Enzyme**	K_{cat}/K_M (min^{-1}M^{-1})
**BT3349$^{4S\text{-}sulf}$(S84C)**	(5.0 ± 0.6) x 10^6
**D33A**	NA
**N105A**	NQ
**R110A**	(9.1 ± 0.4) x 10^3
**R110K**	(2.4 ± 0.22) x 10^5
**W163A**	(1.3 ± 0.01) x 10^3
**W163F**	(7.4 ± 1.3) x 10^2
**T183A**	(3.4 ± 0.2) x 10^6
**D185A**	NQ
**D185N**	NQ
**H241A**	NA
**S242A**	NQ
**D319A**	NA
**H320A**	NQ
**D331A**	NE
**N334A**	NQ
**BT33336$^{S\text{-}sulf}$(S83C)**	Active
**D173A**	Inactive
**R174A**	Inactive
**H223A**	Inactive
**D339A**	Inactive
**W464A**	Active
**Y363A**	Inactive
**Y363F**	Active
**Y363D/D339A**	Inactive
**Y363E/D339A**	Inactive
**BT3348^{GH88}**	
**Δ4,5UA-GalNac**	(2.5 ± 0.09) x 10^5
**Δ4,5UA-GlcNac**	(2.8 ± 0.07) x 10^5
**Δ4,5UA-6SGalNac**	(2.2 ± 0.13) x 10^5
**Δ4,5UA-4S6SGalNac**	NA
**Δ4,5UA2S-6SGalNac**	NA
**Δ4,5UA2S-4S6SGalNac**	NA
**Δ4,5UA-GlcNac (β1,4 linked)**	NA

Supplementary Table 4. Kinetic parameters for periplasmic hydrolase enzymes

For BT3349$^{4S\text{-}sulf}$ the substrate assayed was Δ4,5UA-4S6SGalNac. For BT3333$^{6S\text{-}sulf}$ the substrate assayed was GalNAc6S. NE means the enzyme did not express. NA indicates no activity could be detected; NQ indicates activity could be observed qualitatively by TLC using >10 uM enzyme overnight at 37°C but could not be accurately quantified by spectrophotpmetric methods deployed. All assayed were performed in 100 mM MES pH6.5 with 150 mM NaCl at 37°C. All data were carried out in at least triplicate and ± indicates standard error of the mean. Source data are provided in the source data file labelled as kinetics.

Locus tag<br>Module	BT3324<br>PL8	BT3328<br>PL29	BT3329<br>SGBP	BT3330<br>SGBP	BT3331<br>SusD	BT3332<br>SusC	BT3333<br>Sulf1_15	BT3334<br>HTCS	BT3348<br>GH88	BT3349<br>Sulf1_27	BT3350<br>PL8	BT4410<br>PL33	Growth<br>CS	 DS	 HA	Environment	Taxo class
Bacteroides thetaiotaomicron VPI-5482	100	100	100	100	100	100	100	100	100	100	100	100					
Bacteroides ovatus ATCC 8483	83	38	31	33	61	73	88	90	97	90	86	91					
Bacteroides caccae ATCC 43185	84	38	33	41	60	72	89	87	97	92	83	90					
Bacteroides xylanisolvens XB1A *	83	39	32	34	61	73	89	90	97	90	85	91					
Bacteroides plebeius DSM 17135	67	46	40	40	60	74	74	53	70	73							
Bacteroides cellulosilyticus DSM 14838	71				39	50	79	70	81	82	64	69					
Bacteroides intestinalis DSM 17393	70				38	50	78	70	84	80	65	69					
Bacteroides eggerthii DSM 20697	69				38	51	76	69	82	76	64						
Bacteroides fragilis NCTC 9343									62			57					
Bacteroides vulgatus ATCC 8482									61								
Bacteroides dorei DSM 17855									61								
Bacteroides oleiciplenus YIT 12058	71				39	50	79	70	83	82	65	69					
Bacteroides stercoris ATCC 43183	70				41	50	77	69	84	77	59	38					
Bacteroides clarus YIT 12056	70	40			37	45	76	70	79	78	60	38					
Bacteroides gallinarum DSM 18171	69	40			35	44	76	70	84	77	21	38				chicken gut	
Bacteroides helcogenes P 36-108	66			55	62	69	71	69	81	78		55				pig gut	
Bacteroides fluxus YIT 12057	70				30		76	70	71	79							
Bacteroides uniformis ATCC 8492	69							70	84	81							
Prevotella bergensis DSM 17361	59	34		49	62	64		39	53	68		52				human skin	Bacteroidia
Coprobacter fastidiosus DSM 26242	42			30	39	56	68		72	64	47	60					Bacteroidia
Gabonia massiliensis GM3	60				37	48		60	81	79	26	51					Bacteroidia
Alistipes shahii WAL 8301	41	44	17		33	48			61	56	45	44					Bacteroidia
Pedobacter africanus DSM 12126	37	30		31	40	46	54		49		22	27				soil	Sphingobacteria
Pseudopedobacter saltans DSM 12145	35	28			41	49	56		54		23	32				soil	Sphingobacteria
Niabella soli DSM 19437	26				27	40	53		48			52				soil	Chitiniphagia
Echinicola pacifica DSM 19836		28				36	48		42	36	28	37				sea urchin	Cytophagia

Intermediate growth
Strong growth

*B. xyl BT3334 gene model being missing, identity percentage was based on average 10 other strains

Supplementary Table 5 Conservation across selected *Bacteroidetes* species of the key PUL proteins in *B. thetaiotaomicron*.
Each key PUL proteins is represented as a column headed with *B. theta* locus and functional module tag. Each line represent a species genome with cells indicating the identity percentage of the orthologous proteins (reciprocal best BLAST hit). The eleven species tested for growth are listed on top, with growth results summarised at right. Seven additional *Bacteroides* species are listed in the centre, most selected based on their high conservation levels but additional model species like *B. fluxus* and *B. uniformis* were shown to illustrate unlikely growth ability by a system similar to *B.theta*. Eight non-Bacteroides species are listed at the bottom, with their taxonomic class indicated at right, that displayed high conservation levels of key enzymes, despite important evolutionary distance. The sampling ecosystem is additionally displayed at right, if not "human gut".

	BT3349$^{4S-sulf}$ CSDP4	BT3333$^{6S-sulf}$ GalNAc6S
Beamline	IO-4	I-24
Date	19/02/18	25/01/19
Wavelength (Å)	0.98	0.98
Resolution (Å)	68.89-2.8 (2.92-2.8)	70.55-1.98 (2.03-1.98)
Space group	$P22_12_1$	$P4_12_12$
Unit-cell parameters		
a, b, c (Å)	51.91, 93.91, 304.09	86.38, 86.38, 122.14
α = β = γ (°)	90	90
No. of measured reflections	277,083 (33,576)	467,335 (32,871)
No. of independent reflections	37,857 (4,540)	32,979 (2,253)
Completeness (%)	100 (100)	100 (100)
Redundancy	7.3 (7.4)	14.2 (14.6)
<I>/<σ(I)>	5.6 (1.5)	11.0 (1.5)
CC(1/2)	0.976 (0.543)	0.998 (0.561)
Refinement statistics*		
R_{work}/R_{free} (%)	0.20/0.26	18/22
No. of non-H atoms		
No. of protein, atoms	11,506	3676
No. of solvent atoms	49	112
No. of ligand atoms	126	28
R.m.s. deviation from ideal values		
Bond angle (°)	1.46	1.53
Bond length (Å)	0.006	0.008
Average B factor ($Å^2$)		
Protein	37.49	28.78
Solvent	38.79	27.82
Ligand	53.59	50.63
Ramachandran plot$^+$, most favoured regions (%)	94	96
PDB code	6S21	6S20

Supplementary Table 6. Data collections and refinement statistics.
Values in parenthesis are for the highest resolution shell. R_{free} was calculated using a set (5%) of randomly selected reflections that were excluded from refinement.

Primer	Sequence
BT3324-Forward primer-	ggcatagctagccagttacctgcctgtataaccgtt
BT3324-Reverse primer-	tattccctcgagttatttctccagttctacctcata
BT3328-Forward primer	CGCGGGATCCgacgaacgggatgatctg
BT3328-Reverse primer	CGCGCTCGAGttatttaagtgcgttgagccatc
BT3329-Forward primer	ggcataggatccgatacagacttgagagacgat
BT3329-Reverse primer	tattccctcgagttagttggttgcaccgtattcggc
BT3330-Forward primer	ggcataggatccgacgggctggacgaagcggtaggt
BT3330-Reverse primer	tattccctcgagttattccactacgtttaccaccat
BT3331-Forward primer	ggcataggatccgactttctggacaaagaacctacc
BT3331-Reverse primer	tattccctcgagttaccaacccgggttgttctgtgt
BT3333-Forward primer	ggcatagctagcggatgcaatcagaaagtacaaaaa
BT3333-Reverse primer	tattccctcgagttatcttgtctttaccgattcaag
BT3348-Forward primer	ggcataggatccggtggacaaaaaaaggttgaagtc
BT3488-Reverse primer	tattccctcgagttacttcttttccaggtctctttt
BT3349-Forward primer	ggcatagctagccaaggctgtaaagcccccaagcag
BT3349-Reverse primer	tattccctcgagtcagtaaggtatacggtccgaaag

BT3350-Forward primer	ggcatagctagccaggtcatagggtttgaagaaaag
BT3350-Reverse primer	tattccctcgagtcatttattttcaagaatcatttc
BT4410-Forward primer	ggcatagctagaatgcctatacagaacgcgatatg
BT4410-Reverse primer	attccctcgagctatttaatttgttgaataacaaa
BT3333-D173A-Forward primer	ATTCCGGCAACCGTAGCCCGTGTCCCCTGTGTATTCGTC
BT3333-D173A-Reverse primer	TACACAGGGGACACGGGCTACGGTTGCCGGAATCACGAA
BT3333-R174A-Forward primer	CCGGCAACCGTAGACGCCGTCCCCTGTGTATTCGTCGAG
BT3333-R174A-Reverse primer	GAATACACAGGGGACGGCGTCTACGGTTGCCGGAATCAC
BT3333-H223A-Forward primer	AAACCCAGTCAGGGAGGCAACAATACGATAATCAACGGC
BT3333-H223A-Reverse primer	GATTATCGTATTGTTGGCTCCCTGACTGGGTTTTAGTTT
BT3333-D339A-Forward primer	GGCCCCGTCATCGACGGCGGCTACCAGGACCAAGCCTTT
BT3333-D339A-Reverse primer	TTGGTCCTGGTAGCCGGCGTCGATGACGGGGCCATTGTC
BT3333-Y363A-Forward primer	TACCGTGGTGGAAAGGCCAGCGCTTACGAAGCCGGAACC
BT3333-Y363A-	GGCTTCGTAAGCGCTGGCCTTTCCACCACGGTAAATACC

Reverse primer	
BT3333-Y363F-Forward primer	TACCGTGGTGGAAAGTTTAGCGCTTACGAAGCCGGAACC
BT3333-Y363F-Reverse primer	GGCTTCGTAAGCGCTAAACTTTCCACCACGGTAAATACC
BT3333-Y363D - Forward primer	TACCGTGGTGGAAAGGATAGCGCTTACGAAGCCGGAACC
BT3333-Y363D-Reverse primer	GGCTTCGTAAGCGCTATCCTTTCCACCACGGTAAATACC
BT3333-Y363E-Forward primer	TACCGTGGTGGAAAGGAGAGCGCTTACGAAGCCGGAACC
BT3333-Y363E-Reverse primer	GGCTTCGTAAGCGCTCTCCTTTCCACCACGGTAAATACC
BT3349-D33A-Forward primer	ATCTATGTATTTCCTGCCCAGTATCGCAATCAGGCAATG
BT3349-D33A-Reverse primer	CTGATTGCGATACTGGGCAGGAAATACATAGATGATGTT
BT3349-N105A-Forward primer	AGTGGTGTACCTTTGGCCTGTAACTCTACACGTCCTATC
BT3349-N105A-Reverse primer	ACGTGTAGAGTTACAGGCCAAAGGTACACCACTGCGATT
BT3349-R110A-Forward primer	AACTGTAACTCTACAGCCCCTATCAGCTCTTTGCGTGAC
BT3349-R110A-Reverse primer	CAAAGAGCTGATAGGGGCTGTAGAGTTACAGTTCAAAGG

BT3349-R110K-Forward primer	AACTGTAACTCTACAAAACCTATCAGCTCTTTGCGTGAC
BT3349-R110K-Reverse primer	CAAAGAGCTGATAGGTTTTGTAGAGTTACAGTTCAAAGG
BT3349-W163A-Forward primer	ACACAGCGTCCGGTGGCCGACGCTTATACGCCGAAAGAG
BT3349-W163A-Reverse primer	CGGCGTATAAGCGTCGGCCACCGGACGCTGTGTTTCTAC
BT3349-W163F-Forward primer	ACACAGCGTCCGGTGTTTGACGCTTATACGCCGAAAGAG
BT3349-W163F-Reverse primer	CGGCGTATAAGCGTCAAACACCGGACGCTGTGTTTCTAC
BT3349-T183A-Forward primer	TGGTATTCGTACGGCGCCTTTGACGAACATAAGAACCCG
BT3349-T183A-Reverse primer	CTTATGTTCGTCAAAGGCGCCGTACGAATACCAATAGTT
BT3349-D185A-Forward primer	TCGTACGGCACGTTTGCCGAACATAAGAACCCGCATTAT
BT3349-D185A-Reverse primer	CGGGTTCTTATGTTCGGCAAACGTGCCGTACGAATACCA
BT3349-D185N-Forward primer	TCGTACGGCACGTTTAATGAACATAAGAACCCGCATTAT
BT3349-D185N-Reverse primer	CGGGTTCTTATGTTCATTAAACGTGCCGTACGAATACCA
BT3349-H241A-	GGAATGAACCCGCCGGCCAGCCCATACCGTTCGCTGAAC

Forward primer	
BT3349-H241A-Reverse priemr	CGAACGGTATGGGCTGGCCGGCGGGTTCATTCCCACCAT
BT3349-S242A-Forward primer	ATGAACCCGCCGCATGCCCCATACCGTTCGCTGAACGAT
BT3349-S242-Reverse primer	CAGCGAACGGTATGGGGCATGCGGCGGGTTCATTCCCAC
BT3349-D319A-Forward primer	GTCATCTTTGCTTCCGCCCATGGCGAAACAATGTGCAGC
BT3349-D319A-Reverse primer	CATTGTTTCGCCATGGGCGGAAGCAAAGATGACTACTGT
BT3349-H320A-Forward primer	ATCTTTGCTTCCGACGCCGGCGAAACAATGTGCAGCCAG
BT3349-H320A-Reverse primer	GCACATTGTTTCGCCGGCGTCGGAAGCAAAGATGACTAC
BT3349-D331A-Forward primer	AGCCAGCGAACGGACGCCCCTAAGAACTCTCCCTATTCC
BT3349-D331A-Reverse primer	GGGAGAGTTCTTAGGGGCGTCCGTTCGCTGGCTGCACAT
BT3349-N334A-Forward primer	ACGGACGACCCTAAGGCCTCTCCCTATTCCGAATCTATG
BT3349-N334A-Reverse primer	TTCGGAATAGGGAGAGGCCTTAGGGTCGTCCGTTCGCTG

Supplementary Table 7. Table of primers used in the study.
Cyan, yellow and red highlight BamHI, NheI and XhoI exnzyme restriction sites. Green indicates the site of mutation in mutagenesis primers.
